# Isolation and Microbiological and Molecular Identification of *Brucella abortus* in Cattle and Pigs, Slaughtered in Cattle Sheds Located in Northern Sierra of Ecuador

**DOI:** 10.3390/pathogens14101003

**Published:** 2025-10-03

**Authors:** Maritza Celi-Erazo, Elizabeth Minda-Aluisa, Lisbeth Olmedo-Pinchao, Lenin Ron-Garrido, Tania Ortega-Sierra, Julián López-Balladares, Marlon Carlosama-Yépez, Santiago Gonzalón-Alcarraz, Jacobus H. de Waard, Claude Saegerman, Jorge Ron-Román, Washington Benítez-Ortiz

**Affiliations:** 1 Instituto de Investigación en Zoonosis CIZ, Universidad Central del Ecuador, Quito 170521, Ecuador; sminda@uce.edu.ec (E.M.-A.); lsolmedo@uce.edu.ec (L.O.-P.); ljron@uce.edu.ec (L.R.-G.); santiagogonzalon@gmail.com (S.G.-A.); washobenitez@yahoo.es (W.B.-O.); 2 Grupo de Investigación en Biodiversidad, Salud Pública y Zoonosis (GIBCIZ), Universidad Central del Ecuador, Quito 170521, Ecuador; 3 Facultad de Medicina Veterinaria y Zootecnia, Universidad Central del Ecuador, Quito 170521, Ecuador; tanialex171@gmail.com (T.O.-S.); mvz.julianlopez@gmail.com (J.L.-B.); marlonhcarlosama@hotmail.com (M.C.-Y.); 4 Coordinación General de Registro de Insumos Agropecuarios, Agencia de Regulación y Control Fito y Zoosanitario-AGROCALIDAD, Quito 170518, Ecuador; 5 Dirección de Posgrados, Coordinación de la Maestría en Producción Animal con Mención en Nutrición Animal, Universidad UTE, Quito 170147, Ecuador; 6 One Health Research Group, Facultad de Ciencias de la Salud, Universidad de Las Américas (UDLA), Quito 170125, Ecuador; jacobus.dewaard@udla.edu.ec; 7 Research Unit of Epidemiology and Risk Analysis Applied to Veterinary Sciences (UREAR-ULg), Fundamental and Applied Research for Animals & Health Center, Department of Infections and Parasitic Diseases, Faculty of Veterinary Medicine, University of Liege, 4000 Liege, Belgium; 8 Grupo de Investigación en Salud Animal y Humana (GISAH), Carrera de Ingeniería Agronómica, Departamento de Ciencias de la Vida y Agrícolas, Universidad de las Fuerzas Armadas ESPE, Sangolquí 171103, Ecuador; jwron@espe.edu.ec

**Keywords:** *Brucella* spp., cattle, swine, seropositive, isolation, characterization, IS711 PCR, AMOS PCR, Rose Bengal (RB) test and sero-agglutination test (SAT)-EDTA

## Abstract

Brucellosis remains an underreported zoonotic disease in Ecuador. Its control program in cattle integrates diagnostic testing, vaccination, and eradication incentives, although participation is largely voluntary. Since 2025, vaccination has become compulsory nationwide. Human surveillance remains largely passive, and strain-level data are very limited. This study applied an integrated approach, combining serology (Rose Bengal and SAT-EDTA), microbiological culture, and molecular diagnostics, to assess the presence and diversity of *Brucella* spp. in cattle and pigs from six slaughterhouses in the northern Andean highlands. A total of 2054 cattle and 1050 pigs from Carchi, Imbabura, and Pichincha were sampled. Among cattle, 133 (6.5%; 95% CI: 5.5–7.6) were seropositive, and viable *B. abortus* strains were isolated from 17 (12.8%). Genus identification was confirmed by IS711-PCR, while species- and biovar-level differentiation was achieved with AMOS-PCR; additional assays targeting the *ery* gene and RB51 marker were used to distinguish field from vaccine strains. Biotyping and molecular analysis revealed a predominance of *B. abortus* biovar 4 (13/17 isolates) over biovar 1, all confirmed as field strains. In pigs, 10 animals (0.95%) tested seropositive, but no isolates were recovered, highlighting limitations of serology in swine. Most livestock, including the positives, originated locally, reinforcing the representativeness of our findings. The successful isolation and molecular characterization of *B. abortus* demonstrates the value of combining diagnostic strategies beyond serology. These results underscore the utility of active surveillance when supported by traceability systems; this approach may also contribute to guide interventions to reduce infection risk in livestock and humans.

## 1. Introduction

Brucellosis is a zoonotic disease caused by bacteria of the genus *Brucella*, which are Gram-negative, facultative intracellular coccobacilli infecting a wide range of mammals, including humans and livestock [1]. The disease remains a significant global zoonotic and public health and economic concern due to its impact on reproductive efficiency in animals and the risk of transmission to humans through direct contact or consumption of unpasteurized animal products. In Latin America, brucellosis persists despite control and vaccination programs; this endemicity is driven by factors such as complex smallholder livestock systems, unregulated animal movements, insufficient indemnities and surveillance, and under-resourced veterinary infrastructure [2].

In cattle, *B. abortus* is the species most frequently implicated, whereas *B. suis* predominates in pigs [3]. In cattle, acute infection is often associated with reproductive events, driven in part by the high concentrations of erythritol in placental and reproductive tissues, which strongly favor *Brucella* growth. This stage typically leads to abortion (often in the last trimester), retained placenta, orchitis, and reduced fertility. Chronic infection, by contrast, is characterized by persistent colonization of lymph nodes, mammary glands, and reproductive tissues, often without overt clinical signs, but with continued bacterial shedding and transmission risk. In pigs, acute brucellosis similarly manifests with abortion, infertility, orchitis, and lameness, while chronic cases may result in abscess formation in lymph nodes or reproductive organs, contributing to long-term persistence in herds. These clinical patterns complicate diagnosis and underscore the importance of laboratory confirmation for both surveillance and control. In this context, biovar typing is critical for distinguishing vaccine strains from circulating field isolates, tracing outbreaks, and assessing cross-species transmission. In Argentina, for instance, *B. abortus* biovars 1 and 4 are prevalent in cattle, and atypical *B. suis* strains—often resistant to dyes—have been associated with pigs [4].

In Ecuador, the epidemiological picture is complex and understudied. Although serological studies have indicated a national herd-level seroprevalence of 8% and an individual-level prevalence of 2.2% in dairy cattle [5], and 17% in dual-purpose systems [6]. Brucellosis control is addressed through the National Bovine Brucellosis Control Program, led by Agrocalidad, which integrates epidemiological surveillance, diagnostic testing, vaccination, and eradication incentives. Participation is still largely voluntary, and any herd can obtain “officially brucellosis-free” status through routine testing and culling of reactors; however, dairy herds receive stronger economic incentives (approximately USD 0.01 per liter of milk produced) once free status is achieved. This targeted benefit, absent in beef cattle operations, may partly explain the higher prevalence reported in certain coastal provinces where beef production predominates. Vaccination is a core component of the national bovine brucellosis control program in Ecuador; however, during the period of this study its application was voluntary and often limited to herds enrolled in official eradication schemes. Two vaccines were available: *Brucella abortus* strain S19 and strain RB51 in cattle. Coverage remains low, with estimates suggesting that fewer than 25% of animals are vaccinated nationwide [5]. Swine and in other livestock species vaccination is not practiced. However, as of 2025, vaccination with the *B. abortus* RB51 strain has become compulsory nationwide, specifically targeting bovine and buffalo females from 3 months of age onwards, with a two-dose scheme: the first dose administered between 3 and 9 months of age and a booster between 9 and 15 months (i.e., 6 months after the first). Moreover, strain and biovar characterization are not incorporated into the program, leaving important aspects of bovine brucellosis epidemiology and potential cross-species transmission pathways unknown. Oversight and enforcement limitations, including infrequent inspections, further constrain program effectiveness. Parra et al. (2021) reported molecular detection of *B. abortus* DNA in seropositive cattle from the Ecuadorian highlands, confirming infection of these animals by field strains using PCR assays targeting bcsp31 and omp2a [7]. Meanwhile, Ron-Román et al. (2019) documented regional seroprevalence and associated risk factors in Imbabura, Carchi, and Pichincha provinces—highlighting significant exposure in both cattle and farm workers and underscoring the need for intensified surveillance in these areas [8].

Regarding cattle movement, it clearly plays an important role in the spread of brucellosis. Network analyses of cattle movements (2017–2018) showed that over 90% of parishes are highly connected, with very low fragmentation, indicating frequent movement of animals between regions. Brucellosis, therefore, is likely to persist and circulate within local clusters of herds [9]. Although cattle transhumance (i.e., seasonal migration of cattle for grazing) is very limited in Ecuador, the smallholder systems common in the Andean region increase opportunities for transmission, as animals often share grazing areas and bulls are rented for natural mating. Information on the role of animal movements in other livestock species remains scarce, which limits a broader understanding of transmission dynamics.

Another relevant study in Santo Domingo de los Tsáchilas province identified *B. abortus* biovar 1 as the predominant field strain in cattle, combining classical biotyping with molecular assays, including IS711-PCR for genus confirmation, AMOS-PCR for species and biovar differentiation, and HOOF-Prints MLVA for high-resolution strain typing [10]. However, comprehensive biotyping studies involving other species, such as pigs (the major mammal consumed in Ecuador) and other livestock species, are lacking. Swine production systems in this Andean region range from smallholder backyard operations to semi-intensive farms, and many pigs have free-ranging access to other livestock species such as cattle, or to areas where contact with wildlife (e.g., feral pigs) can occur. This interface between domestic swine and other animal populations represents a potential pathway for cross-species transmission that remains poorly investigated in Ecuador.

Experiences from neighboring countries such as Costa Rica show the added value of incorporating strain-level diagnostics into national strategies. Hernández-Mora et al. (2017) characterized *Brucella* isolates from multiple species and identified *B. abortus* and *B. suis* biovars circulating in cattle and pigs [11]. Their work demonstrates the utility of combining molecular and classical techniques to strengthen public health risk assessments and to detect gaps in surveillance systems.

Molecular tools targeting conserved genes (e.g., *bcsp*31, *omp*2, *IS*711), coupled with classical bacteriology and biotyping, remain indispensable for identifying circulating strains and differentiating field from vaccine strains [12,13,14]. These methods are particularly important in areas where brucellosis remains endemic, and transmission dynamics are not well understood.

Therefore, the aim of this study was to isolate and characterize *Brucella* spp. strains from cattle and pigs sampled at slaughterhouses in the northern Andean provinces of Ecuador (Pichincha, Imbabura, and Carchi). Since isolation success is strongly associated with seropositivity, we focused on seropositive animals to recover bacterial isolates and generate strain- and biovar-specific data, thereby contributing to the understanding of local transmission pathways and supporting more targeted and effective brucellosis control efforts in Ecuador.

## 2. Materials and Methods

### 2.1. Description and Location of the Study

**Figure 1:**
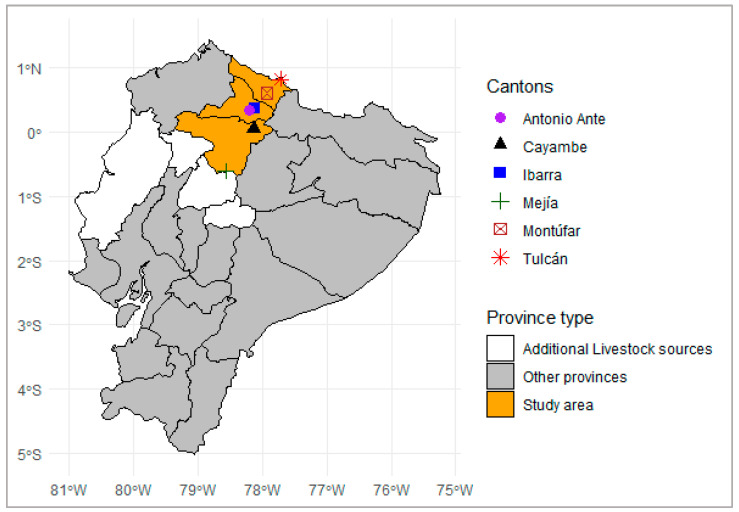
Location of slaughterhouses, and area of origin of sampled cattle and pigs.

Between 2013 and 2017, several field visits were conducted to municipal slaughterhouses in the northern Ecuadorian provinces of Pichincha (*n* = 2; cantons: Mejía and Cayambe), Imbabura (*n* = 2; cantons: Ibarra and Antonio Ante), and Carchi (*n* = 2; cantons: Tulcán and Montúfar). Figure 1 shows the study area and the geographical distribution of the sampled slaughterhouses. The distribution of cattle sampled by province was as follows: Pichincha (32.13%, *n* = 660), Carchi (25.12%, *n* = 516), and Imbabura (22.54%, *n* = 463). Additional samples were obtained from cattle originating in other Andean provinces of Ecuador, including Cotopaxi (1.4%, *n* = 29), Tungurahua (1.3%, *n* = 27), and Cañar (0.1%, *n* = 2), as well as from the coastal provinces of Manabí (12.61%, *n* = 259) and Santo Domingo de los Tsáchilas (4.7%, *n* = 96). In swine, samples originated primarily from Carchi (53.90%; 566/1050), followed by Imbabura (34.76%; 365/1050) and Pichincha (11.33%; 119/1050), all of which are located within the defined study area.

During slaughter, blood samples were collected from the jugular vein using 100 mL sterile bottles. Additionally, tissue samples—including liver, spleen, and retromammary lymph nodes—were aseptically collected from the same animals. All samples were placed in sterile containers and transported under refrigeration (4 °C) to the laboratory for further analysis. A sampling protocol was applied whereby one animal was sampled for every five slaughtered. A total of 2054 cattle (including *Bos taurus* and *Bos indicus*) and 1050 pigs were sampled. Only the Mejia slaughterhouse did not process pigs during the sampling period.

For each sampled animal, a record form was completed to collect zootechnical and traceability data, including age, sex, breed, and geographic origin. This information was used to support the epidemiological analysis and spatial distribution of *Brucella* infection in the region.

### 2.2. Serological Tests for the Diagnosis of Brucellosis

Serological screening was performed using the Rose Bengal Test (RBT) and the Slow Tube Agglutination Test in the presence of EDTA (SAT-EDTA). To optimize laboratory resources and increase the likelihood of isolating *Brucella*, only animals that tested positive in at least one of the two serological tests were selected for bacteriological culture and strain isolation. Although tissue and lymph node samples were collected from all animals, isolation was conducted exclusively on seropositive animals. We acknowledge that this design does not allow detection of *Brucella* in seronegative animals, which could theoretically harbor the pathogen, but seropositive animals increase the likelihood of recovering *Brucella* from the tissues.

For the RBT, the Pourquier^®^ Rose Bengal antigen (IDEXX, Westbrook, ME, USA), derived from *Brucella abortus* Weybridge 99 strain, was used, following the World Organisation for Animal Health (WOAH, formerly OIE) standard protocol. Similarly, the SAT-EDTA test utilized antigen prepared from the same *B. abortus* Weybridge 99 strain, in line with WOAH/OIE recommendations. Sera were classified as positive based on visible agglutination, indicating the presence of *Brucella*-specific antibodies. Agglutination intensity was graded as follows: negative (−), low (+), moderate (++), high (+++), and very strong (++++) responses.

For the SAT-EDTA, the antigen was also obtained from IDEXX (Westbrook, ME, USA), and the procedure followed the protocol established by the Brucellosis Reference Laboratory of Belgium. A cut-off value of 30 International Agglutination Units (IAU)—equivalent to 25% agglutination at a 1:25 dilution—was used for both cattle and pig sera.

Samples testing positive by either or both tests were classified as seropositive under a parallel testing approach. The combined use of RBT and SAT-EDTA enables the detection of antibodies across both acute and chronic phases of infection, improving the diagnostic sensitivity of the screening protocol.

### 2.3. Isolation and Biotyping of the Brucella Species

Organ and lymph node samples from seropositive animals were individually macerated using a Stomacher^®^ homogenizer. Each macerate was cultured on Farrell’s selective medium, consisting of Columbia Blood Agar Base (CM0331, Oxoid, Basingstoke, United Kingdom) supplemented with 5% heat-inactivated horse serum (Ref. 16050–130, Gibco, New Zealand) and modified *Brucella* selective supplement (SR0209E, Oxoid, Basingstoke, United Kingdom). Inoculated plates were incubated at 37 °C with 5% CO_2_ for up to 4 weeks.

Colonies suspected to be *Brucella* spp. were subjected to a panel of microbiological and biochemical tests for confirmation and biotyping. Preliminary identification included colony morphology, Gram staining, and catalase activity. Species and biovar identification were carried out based on oxidase activity, hydrogen sulfide (H_2_S) production, urease activity, carbon dioxide (CO_2_) requirement, and agglutination with monospecific anti-A (Ref. R30164801 ZM01, Remel, Dartford, United Kingdom) and anti-M (Ref. R30164901 ZM02, Remel, Dartford, United Kingdom) sera specific for *Brucella*, which were used to confirm the antigenic expression of epitopes A and M.

Additionally, growth inhibition on media containing selective dyes (basic fuchsin, thionine, and safranin) was used for biovar differentiation, following established protocols for *Brucella* species classification [15]. Confirmed *Brucella* isolates were preserved at –80 °C in cryotubes containing glycerol-based cryopreservative.

For subsequent molecular analysis, 2–3 colonies from each isolate were suspended in 1.5 mL microtubes containing sterile distilled water. The suspensions were heat-inactivated at 65 °C, and their sterility was verified by subculturing onto Columbia Blood Agar to ensure the absence of viable organisms before DNA extraction and PCR testing.

### 2.4. Molecular Identification and Typing of Brucella spp.

For molecular identification, three PCR-based assays were performed to confirm genus, determine species, and differentiate vaccine strains from field isolates. Firstly, DNA extraction was performed from each *Brucella*-positive isolate. Bacterial suspensions were centrifuged at 2300× *g* for 10 min, and the resulting pellets were resuspended in TE buffer (10 mM Tris-HCl, 1 mM EDTA, pH 8.0). A thermal lysis procedure consisting of two freeze–thaw cycles (65 °C/60 min) was applied. Supernatants were recovered by centrifugation and stored at −20 °C until further analysis.

Genus-level identification was achieved using IS711-PCR (also referred to as IS6501-PCR), targeting the IS711 insertion sequence, which is conserved among *Brucella* species and enables differentiation from other α-Proteobacteria. Amplification of a 261 bp product confirmed genus identification [16,17].

Species-level identification was conducted using the AMOS-PCR method [13], which allows multiplex detection of *B. abortus* biovars 1, 2, and 4 (498 bp); *B. melitensis* biovars 1–3 (731 bp); *B. ovis* (976 bp); and *B. suis* biovar 1 (285 bp). Reactions were performed with species-specific primers and known DNA controls.

To differentiate vaccine strains from field isolates, a PCR targeting the *ery* gene was performed. A 178-bp fragment, present in field strains but absent in the *B. abortus* S19 vaccine strain, was used as a marker. In addition, the absence of a 364-bp fragment specific to the RB51 vaccine strain was evaluated [18,19]. Amplifications were performed in a 20 μL reaction volume containing 1 U of Taq DNA polymerase, 1× buffer, 1.5 mM MgCl_2_, 0.2 mM of each dNTP, 0.2 μM of each primer, and approximately 10 ng of DNA template.

Primer sequences used for all PCR assays are provided in Table 1.

### 2.5. Data Analysis

All data were organized using Microsoft Excel^®^ and subsequently analyzed in the R environment (version 4.1.1). Seroprevalence estimates and their 95% confidence intervals were calculated under the assumption of simple random sampling for both cattle and pigs. Chi-square tests were used to assess associations between sex and seropositivity, as well as to compare seropositivity proportions among provinces. In addition, the Kappa coefficient was calculated to evaluate the level of agreement between the RB and SAT-EDTA serological tests. Statistical significance was set at *p* < 0.05.

## 3. Results

### 3.1. Bovine Testing Results

#### 3.1.1. Serological Screening of Bovines

As summarized in Table 2, a total of 2054 cattle were serologically tested for brucellosis. Of these, 4.9% (101/2054) tested positive using the Rose Bengal Test (RBT), and 6.1% (126/2054) were positive using SAT-EDTA. When considering animals that tested positive in either or both tests (parallel testing), the estimated overall seroprevalence of brucellosis in cattle was 6.5% (135/2054; 95% CI: 5.5–7.6%). A high level of agreement was observed between the two serological tests, with a Kappa coefficient of 0.82 (95% CI: 0.78–0.86; *p* < 0.05), indicating strong concordance between RBT and SAT-EDTA in identifying seropositive animals.

Detailed data on the number of animals sampled, their provincial origin (only the origin of two animals was missing), and their serological and microbiological results are shown in Table 2. Notably, approximately 80% of the slaughtered cattle originated from the direct geographical area covered by this study. In the same way, 88.1% (119/135; 95% CI: 81.2–93.0%) of the seropositive animals originated from the study area itself, further supporting the local circulation and endemic presence of *Brucella* strains in the region.

#### 3.1.2. Isolation and Biotyping of *Brucella* Isolates

A total of 17 isolates were obtained and identified as *Brucella* spp., representing an isolation rate of 12.6% (95% CI: 7.8–19.7%) from a total of 135 samples (Table 3). Among the different tissue samples analyzed, retromammary lymph nodes yielded the highest number of positive isolates, accounting for 76.47% (13/17) of all confirmed cases. Microbiological and biochemical characterization allowed for further typing of the isolates. *B. abortus* biovar 1 was identified in 4 out of 17 isolates (23.53%), while *B. abortus* biovar 4 was the predominant biovar, detected in 13 out of 17 isolates (76.47%).

Table 2 summarizes the geographical distribution of *Brucella* isolates and the origin of the sampled animals. Statistically significant differences were found in the proportion of isolates by sex (*p* < 0.05), with females representing 94.12% (16/17) of positive cases, compared to 5.88% (1/17) from males. Similarly, significant differences were observed in the distribution of isolates by slaughterhouse location (*p* < 0.05), with the highest number reported in Pichincha (58.82%, 10/17), followed by Carchi (29.41%, 5/17), and Imbabura (11.76%, 2/17). With respect to the province of origin of the animals, 47.06% of isolates (*n* = 8) came from Pichincha, 29.41% (*n* = 5) from Carchi, 17.65% (*n* = 3) from Imbabura, and 5.89% (*n* = 1) from Manabí.

Notably, isolate 14—originally classified as *B. abortus* biovar 4—exhibited atypical behavior in biochemical tests. Unlike the reference strain for this biovar, isolate 14 was not inhibited by Basic Fuchsin (20 µg) or Safranin (100 µg), suggesting a possible variant. Table 4 presents the results of microbiological and biochemical tests for identification and typing of *Brucella* spp. in cattle.

#### 3.1.3. Molecular Identification

**Figure 2:**
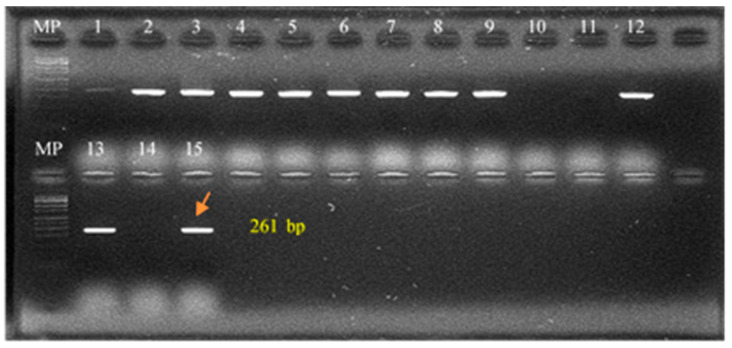
Representative results of the IS711 PCR assay. PCR products were separated on a 2% agarose gel and stained with ethidium bromide. MP: 100-bp molecular weight marker. Lanes 1–9: field samples analyzed in this study. Lanes 10, 11, and 14: negative controls. Lanes 12, 13, and 15: positive controls (*Brucella abortus*). The arrow indicates the expected 261-bp fragment specific to the genus *Brucella*.

**Figure 3:**
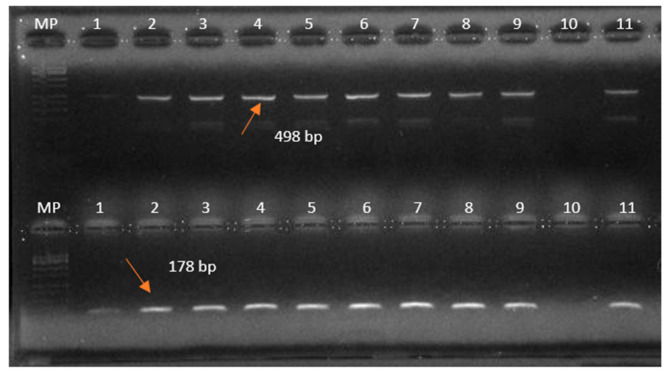
Products of AMOS-PCR and ery-PCR assays. PCR amplicons were electrophoresed on a 2% agarose gel and visualized with ethidium bromide staining. The AMOS-PCR assay, designed to identify the *Brucella* species, shows the characteristic 498 bp amplicon of *B. abortus* in the positive control (lane 11) and in test samples (lanes 1–9). At the bottom products of *ery*-PCR assay specific for field strains shows the corresponding 178 bp fragment in the same test samples, this fragment is absent in S19 and RB51 vaccine strains. MP, 100 bp molecular weight marker; lanes 1–9 sample of this study; lane 10, negative control; The arrow points to the 498 bp fragment corresponding to *B. abortus* and 178 bp to the *ery* locus.

Table 5 shows the results of the molecular tests performed on the 17 *Brucella* spp. iso-lates from cattle. Complete concordance with microbiological typing was observed. Figure 2 shows the products of the IS711 PCR assay, which revealed a 261-bp band characteristic of the genus *Brucella*. Figure 3 presents the results of the AMOS-PCR assay, demonstrating a 498-bp fragment typical of *B. abortus*. In the same assay, PCR targeting the *ery* gene and RB51-specific fragments allowed the differentiation between field and vaccine strains of *B. abortus*. These results confirmed that the *B. abortus* biovar 1 isolates detected in this study were field strains, and that no live vaccine strains were circulating. Although the S19 vaccine strain was not included as a control, the expected fragment sizes for *B. abortus* vaccines are well documented in the literature.

### 3.2. Swine Testing

A total of 1050 swine blood samples were analyzed using both the Rose Bengal (RB) and Serum Agglutination Test with EDTA (SAT-EDTA) assays. Table 6 presents the distribution of sampled animals across slaughterhouses, the number of seropositive results per location, and the province of origin of the pigs.

Of the total samples, 11 animals tested positive by serology, distributed as follows: Carchi (*n* = 2), Imbabura (*n* = 4), and Pichincha (*n* = 5). The estimated overall seroprevalence was 1.0% (95% CI: 0.00–2.0%). The level of agreement between the RB and SAT-EDTA tests was low and not statistically significant, with a Kappa coefficient of 0.15 (95% CI: 0.09–0.20; *p* > 0.05).

None of the 11 lymph nodes and internal organ samples from seropositive swine yielded *Brucella* spp. isolates.

## 4. Discussion

This study aimed to identify and characterize *Brucella* spp. in cattle and pigs from slaughterhouses located in three provinces of the northern Ecuadorian highlands (Carchi, Imbabura, and Pichincha). It is the first study that combines serological screening, classical microbiology, biovar typing, and molecular diagnostics to assess *Brucella* infection dynamics across two livestock species in this region. Our findings provide important insights for the national brucellosis control program and offer baseline data for strain-level surveillance in Ecuador.

### 4.1. Brucellosis in Cattle

Using serology (RB and SAT-EDTA), we observed an overall seroprevalence of 6.5% in cattle. This value is consistent with previously reported estimates in Ecuador [5,6], reinforcing the endemic nature of bovine brucellosis in the region. The high agreement but not perfect between the two tests (κ = 0.82; 95% CI: 0.78–0.86) confirms their complementary diagnostic value: SAT-EDTA is more sensitive in detecting acute infections, while the RB test is better suited to chronic or post-vaccinal exposure [20].

Although the study was not designed to estimate national prevalence due to potential interference from vaccination (e.g., S19 or RB51), these findings should be interpreted in the context of Ecuador’s national brucellosis control program. As described, brucellosis control in Ecuador has historically relied on voluntary participation, with limited vaccination coverage and inconsistent enforcement. Although compulsory nationwide vaccination with *B. abortus* RB51 was only introduced in 2025, its effectiveness and coverage are still under evaluation. The detection of seropositive animals in slaughterhouses therefore reflects the epidemiological situation during the pre-compulsory vaccination period. Biotyping and molecular analysis revealed a predominance of *B. abortus* biovar 4 over biovar 1 in animals originating from the study area, and all isolates were confirmed as field strains, indicating that no live vaccine strains were circulating. Importantly, since most slaughtered animals analyzed in this work were sourced from the defined study area, these results are representative of the local epidemiological situation in this part of the Ecuadorian highlands rather than of the country as a whole. Overall, our study provides locally relevant evidence that complements official surveillance, highlights persistent gaps in herd-level control, and offers a baseline reference against which the future impact of compulsory RB51 vaccination can be assessed.

*Brucella* isolation was achieved in 12.6% (17/135) of seropositive cattle. This relatively low culture positivity is likely multifactorial. Our study specifically selected seropositive animals, independent of their pregnancy or parturition status. This approach could have increased the likelihood of isolation, but it might also not have had a substantial effect since many slaughtered animals were non-pregnant females or males, in which *Brucella* load in target tissues tends to be lower; further, Farrell’s medium (though widely used) has limited sensitivity for certain *B. abortus* strains contributing to reduced isolation rates as *Brucella* multiplies most in the reproductive tract. Additionally, acute serological responses perform better around parturition or abortion [21,22]. Notably, 76.5% of successful isolations were obtained from retromammary lymph nodes, supporting previous findings on tissue tropism related to erythritol metabolism and reproductive tract affinity [23,24].

The predominance of *B. abortus* biovar 4 (76.5%) over biovar 1 (23.5%) aligns with previous findings in the region [8,10]. Interestingly, biovar 1 was previously identified in cattle from the coastal province of Santo Domingo de los Tsáchilas [10]. In the current study, similar strains were detected, but now in livestock originating mainly from the same study area in highlands provinces of Pichincha, Carchi, and Imbabura, suggesting either a regional spread of these lineages or the persistence of common circulating strains across ecological zones. This underscores the need for continuous and geographically representative strain monitoring. It is a worth to mention that one isolate (Code 14) displayed atypical growth patterns in the presence of selective dyes, indicating a possible variant within biovar 4, as previously described in strains from Canada and the USA [25].

Brucellosis vaccination in Ecuador uses *B. abortus* strains S19 and RB51, with vaccination historically voluntary; but RB51 vaccine has become compulsory only in 2025. Our study identified circulating field strains. These findings suggest that the observed seroprevalence largely reflects natural infections rather than vaccine-induced antibodies, so that biovar-specific results highlight local transmission dynamics and underscore the value of molecular surveillance to distinguish field strains from vaccine strains.

### 4.2. Molecular Characterization

The molecular assays confirmed genus and biovar identification, with 100% agreement between phenotypic and genotypic typing. IS711-PCR confirmed all isolates as *Brucella* spp., while AMOS-PCR identified *B. abortus* biovars 1 and 4. To differentiate field strains from vaccine strains (S19 or RB51), we used PCR amplification of the *ery* gene (178 bp), which confirmed that all isolates were field strains, consistent with prior work [18,26]. While RB51 can also be identified using its rough phenotype and resistance to rifampicin, these simpler and cost-effective tests can complement molecular surveillance. In Ecuador, RB51 vaccination is now mandatory, but some farmers still use S19; therefore, integrating molecular detection with phenotypic monitoring is important for accurate surveillance and for distinguishing vaccine-related infections from field infections. These findings highlight the need for improved molecular surveillance and standardized vaccination practices as part of Ecuador’s national control strategy.

### 4.3. Brucellosis in Swine

This study provides the first evidence of *Brucella* seropositivity in pigs in Ecuador. Although the estimated seroprevalence was low (1.0%), the result is epidemiologically relevant, especially given the increasing recognition of swine as potential reservoirs of zoonotic *Brucella* spp. in Latin America [4,11]. The agreement between RB and SAT-EDTA tests was poor, probably due to the low number of positive samples; additionally, no *Brucella* spp. were isolated from tissue samples. These findings raise questions about the specificity of the serological tests in swine. Cross-reactivity of serological tests with *Yersinia enterocolitica* O:9, a common infection in pigs, may explain the seropositive results [27,28]. Given the shared O-polysaccharide antigen between *Y. enterocolitica* and *Brucella* spp., serological testing in pigs should be interpreted with caution. The absence of culture-positive samples in this study limits our ability to confirm true infections. Thus, we emphasize the need to evaluate alternative diagnostic approaches and improve test specificity for porcine brucellosis surveillance [29]. Additionally, the close contact between pigs and cattle, other livestock species in small-scale farming systems in Ecuador facilitates the potential transmission of *Brucella* spp. between species. *Brucella suis* biovar 1 has been reported in neighboring countries such as Colombia and Brazil [4], highlighting the potential for regional spread [29]. These findings underscore the importance of comprehensive surveillance and control measures in pigs and at the livestock-wildlife interfaces.

### 4.4. Value of Active Surveillance in Slaughterhouses

Our findings highlight the strategic value of slaughterhouse-based active surveil-lance. The ability to sample large numbers of animals from diverse geographical origins provides a unique opportunity to detect infected animals that may not show clinical signs. When paired with robust traceability data, such surveillance can help map transmission patterns and inform targeted interventions. Moreover, slaughterhouses represent high-risk points for occupational exposure. We observed infected animals being processed in facilities with inadequate biosafety implementation, reinforcing the need for improved sanitary protocols and routine health monitoring of slaughterhouse personnel [30].

## 5. Conclusions

This study confirms the circulation of *Brucella abortus* biovars 1 and 4—predominantly biovar 4—in cattle from the northern Andean highlands of Ecuador, with a 6.5% seroprevalence detected using RB and SAT-EDTA tests. Seventeen field strains were successfully isolated and molecularly characterized, none corresponding to vaccine strains. Although seropositivity was detected in 11 pigs, no isolates were recovered, marking the first report of seropositive swine in Ecuador, and highlighting the need for improved diagnostic specificity in this species. Given that approximately 80% of the cattle and nearly all the pigs originated from the study area, these results are representative of the local livestock population. The findings underscore the value of slaughterhouse-based active surveillance and traceability as practical tools for monitoring brucellosis, guiding control strategies, and mitigating zoonotic risk.

## 6. Study Limitations

This study was limited to three provinces in the northern Ecuadorian highlands; thus, the findings may not reflect the situation in other regions. While a relatively high seroprevalence was observed in cattle, the isolation rate of *Brucella* spp. was low—likely due to the study design targeting slaughterhouses and probably to use of a single culture medium. To increase the likelihood of successful isolation, we focused on seropositive animals, as isolation is more efficient from this group; however, this approach limited our ability to assess culture outcomes among seronegative individuals, which should be included in future studies, and in particular for prevalence estimations and surveillance activities. Also, this would have allowed us to detect possible subclinical carriers or individuals in early stages of infection, thus broadening the epidemiological picture. Future studies should incorporate multiple selective media (for instance CITA medium) and milk samples collected at different time points, and the inclusion of both *B. suis* biovar 1 (already reported in Colombia and Brazil) and biovar 2 (recognized in European wildlife and swine) in epidemiological surveillance efforts. Additionally, the failure to isolate *Brucella* from seropositive swine raises questions about potential false positives serological reactions due to cross-reactivity; further research is needed to refine diagnostic tools for pigs.

Likewise, the samples analyzed in this study were collected between 2013 and 2017. While the results provide valuable information on circulating *Brucella* strains during that period, the current prevalence and epidemiological patterns may differ due to changes in vaccination coverage, management practices, or control measures. This temporal limitation should be considered when extrapolating the findings to present-day conditions, and ongoing surveillance is recommended to monitor shifts in prevalence and strain circulation.

## Figures and Tables

**Table 1:** Primers used for the molecular typing of *Brucella* spp.

Primer	Sequence (5′–3′)	Length
IS6501 3′	GATAGAAGGCTTGAAGCTTGCGGAC	260 bp
IS6501 5′	ACGCCGGTGTATGGGAAAGGCTTTT	
IS711-specific	TGCCGATCACTTAAGGGCCTTCAT	
*B. abortus*—specific	GACGAACGGAATTTTTCCAATCCC	498 bp
*B. melitensis*—specific	AAATCGCGTCCTTGCTGGTCTGA	731 bp
*B. ovis*—specific	CGGGTTCTGGCACCATCGTCG	976 bp
*B. suis*—specific	GCGCGGTTTTCTGAAGGTTCAGG	285 bp
RB51/2308	CCCCGGAAGATATGCTTCGATCC	364 bp
*eri* 1	GCGCCGCGAAGAACTTATCAA	178 bp
*eri* 2	CGCCATGTTAGCGGCGGTGA	

**Table 2:** Distribution of the serological results (RB and SAT-EDTA) and isolates of *Brucella* spp. in cattle, according to slaughterhouse location and bovine origin.

Slaughterhouse Localization	Slaughterhouse	Province of Animal Origin	Sex of Animals Sampled	Bovines Sampled	Positive Serological Results				Microbiological Isolates	*B. abortus* bv 1	*B. abortus* bv 4
					RB	SAT-EDTA	RB and SAT-EDTA *	Positives to Serology			
Carchi				498							
	Montúfar			245							
		Carchi		245							
			Female	98	0	2	7	9	4	1	3
			Male	147	2	2	1	5	0		
	Tulcán			253							
		Carchi		253							
			Female	153	0	2	8	10	1	1	
			Male	100	0	0	0	0	0		
Imbabura				290							
	Antonio Ante			119							
		Imbabura		119							
			Female	79	0	3	13	16	0		
			Male	40	0	0	0	0	0		
	Ibarra			171							
		Carchi		16							
			Female	1	0	0	0	0	0		
			Male	15	0	0	0	0	0		
		Imbabura		155							
			Female	35	0	2	2	4	0		
			Male	120	0	5	0	5	2		2
Pichincha				1266							
	Cayambe			313							
		Carchi		2							
			Female	2	0	0	0	0	0		
			Male	0	0	0	0	0	0		
		Imbabura		189							
			Female	142	0	4	0	4	1		1
			Male	47	0	0	0	0	0		
		ND		2							
			Female	1	0	0	0	0	0		
			Male	1	0	0	0	0	0		
		Pichincha		120							
			Female	95	0	3	3	6	0		
			Male	25	0	0	0	0	0		
	Mejía			953							
		Cañar		2							
			Female	0	0	0	0	0	0		
			Male	2	0	0	0	0	0		
		Cotopaxi		29							
			Female	25	0	0	2	2	0		
			Male	4	0	0	0	0	0		
		Manabí		259							
			Female	240	0	2	3	5	1		1
			Male	19	0	0	1	1	0		
		Pichincha		540							
			Female	406	4	7	45	56	7	2	5
			Male	134	2	2	0	4	1		1
		Santo Domingo		96							
			Female	87	0	0	5	5	0		
			Male	9	1	0	0	1	0		
		Tungurahua		27							
			Female	9	0	0	2	2	0		
			Male	18	0	0	0	0	0		
**Total**				**2054**	**9**	**34**	**92**	**135**	**17**	**4**	**13**

* This column refers to the number of samples where both tests were positive.

**Table 3:** Zootechnical information and origin of bovines with positive isolates for *Brucella* spp.

Sample Code	Zootechnical Information			Sampling Area and Animal Origin			Serological Test Results		Species and Biovar
	Age (Months)	Sex	Race	Province Location Slaughterhouse	Slaughterhouse	Province of Animal Origin	RB	SAT-EDTA (IAU)	
1	48	M	Taurus	Pichincha	Machachi	Pichincha	+	15	*B. abortus* bv 4
2	66	F	Taurus	Pichincha	Machachi	Pichincha	+	5120	*B. abortus* bv 1
3	30	F	Taurus	Pichincha	Machachi	Pichincha	+	800	*B. abortus* bv 4
4	60	F	Taurus	Pichincha	Machachi	Pichincha	+	3200	*B. abortus* bv 4
5	48	F	Taurus	Pichincha	Machachi	Pichincha	+	800	*B. abortus* bv 4
6	48	F	Taurus	Pichincha	Machachi	Pichincha	+	1600	*B. abortus* bv 1
7	42	F	Taurus	Pichincha	Machachi	Pichincha	+	800	*B. abortus* bv 4
8	72	F	Taurus	Pichincha	Machachi	Pichincha	+	200	*B. abortus* bv 4
9	48	F	Taurus	Pichincha	Machachi	Manabí	−	50	*B. abortus* bv 4
10	54	F	Taurus	Pichincha	Cayambe	Imbabura	−	100	*B. abortus* bv 4
11	24	M	Taurus	Imbabura	Ibarra	Imbabura	−	80	*B. abortus* bv 4
12	18	M	Taurus	Imbabura	Ibarra	Imbabura	−	40	*B. abortus* bv 4
13	60	F	Taurus	Carchi	San Gabriel	Carchi	++++	800	*B. abortus* bv 4
14	72	F	Taurus	Carchi	San Gabriel	Carchi	++++	3200	*B. abortus* bv 4
15	42	F	Taurus	Carchi	Tulcán	Carchi	++++	3200	*B. abortus* bv 1
16	60	F	Taurus	Carchi	San Gabriel	Carchi	+++	240	*B. abortus* bv 1
17	54	F	Taurus	Carchi	San Gabriel	Carchi	++++	6400	*B. abortus* bv 4

IAU: International Agglutination Units; SAT-EDTA cut-off value of 30 IAU; RB: Rose Bengal with intensity of agglutination graded as follows: negative (−), low (+), moderate (++), high (+++), and very strong (++++) responses.

**Table 4:** Results of microbiological tests for identification and typing of *Brucella* spp. in cattle.

Code		Microbiological Test Results					Growth Inhibition on Colorants				Agglutination with Serum		Species and Biovar
	Organ	Catalase	Oxidase	Urease Activity	H_2_S Production	CO_2_ Requirement	Thionin 20 µg	Thionin 10 µg	Basic Fuschin 20 µg	Safranin 100 µg	Anti A	Anti M	
1	Liver	+	+	+	+	+	−	−	+	+	−	+	*B. abortus* bv 4
2	Lymph nodes	+	+	+	+	+	−	−	+	+	+	−	*B. abortus* bv 1
3	Lymph nodes	+	+	−	+	+	−	−	+	+	−	+	*B. abortus* bv 4
4	Lymph nodes	+	+	−	+	+	−	−	+	+	−	+	*B. abortus* bv 4
5	Lymph nodes	+	+	+	+	+	−	−	+	+	−	+	*B. abortus* bv 4
6	Lymph nodes	+	+	+	+	+	−	−	+	+	+	−	*B. abortus* bv 1
7	Lymph nodes	+	+	−	+	+	−	−	+	+	−	+	*B. abortus* bv 4
8	Lymph nodes	+	+	+	+	+	−	−	+	+	−	+	*B. abortus* bv 4
9	Liver, spleen, lymph nodes	+	+	+	+	+	−	−	+	+	−	+	*B. abortus* bv 4
10	Liver	+	+	+	+	+	−	−	+	+	−	+	*B. abortus* bv 4
11	Spleen	+	+	+	+	+	−	−	+	+	−	+	*B. abortus* bv 4
12	Liver	+	+	+	+	+	−	−	+	+	−	+	*B. abortus* bv 4
13	Lymph nodes	+	+	+	+	+	−	−	+	+	−	+	*B. abortus* bv 4
14	Lymph nodes	+	+	+	+	+	−	−	− ^a^	−	−	+	*B. abortus* bv 4
15	Lymph nodes, spleen	+	+	+	+	+	−	−	+	+	+	−	*B. abortus* bv 1
16	Lymph nodes, spleen	+	+	+	+	+	−	−	+	+	+	−	*B. abortus* bv 1
17	Lymph nodes	+	+	+	+	+	−	−	+	+	−	+	*B. abortus* bv 4
C-B2		+	+	+	+	+	−	−	−	−	+	−	*B. abortus* bv 2
C-B9		+	+	+	−	+	+	+	+	+	−	+	*B. abortus* bv 9
C-B1		+	+	+	+ ^a^	+	−	−	+	+	+	−	*B. abortus* bv 1
C-B4		+	+	+	+	+	−	−	+ ^a^	+	−	+	*B. abortus* bv 4

+ = trait detected (biochemical activity or agglutination) or growth inhibited by dye; − = trait absent or no inhibition. C-B1, C-B2, C-B4, and C-B9 are Control *Brucella abortus* biovar 1, 2, 4 and 9, respectively. ^a^ Some strains isolated in Canada, Great Britain and the USA are inhibited by basic fuchsin.

**Table 5:** Results of molecular tests of *Brucella* spp. isolates in cattle.

Sample Code	Molecular Test Results		
	PCR IS711	AMOS PCR	mAMOS PCR
1	*Brucella* spp.	*B. abortus* bv. 4	NP
2	*Brucella* spp.	*B. abortus* bv. 1	Field strain
3	*Brucella* spp.	*B. abortus* bv. 4	NP
4	*Brucella* spp.	*B. abortus* bv. 4	NP
5	*Brucella* spp.	*B. abortus* bv. 4	NP
6	*Brucella* spp.	*B. abortus* bv. 1	Field strain
7	*Brucella* spp.	*B. abortus* bv. 4	NP
8	*Brucella* spp.	*B. abortus* bv. 4	NP
9	*Brucella* spp.	*B. abortus* bv. 4	NP
10	*Brucella* spp.	*B. abortus* bv. 4	NP
11	*Brucella* spp.	*B. abortus* bv. 4	NP
12	*Brucella* spp.	*B. abortus* bv. 4	NP
13	*Brucella* spp.	*B. abortus* bv. 4	NP
14	*Brucella* spp.	*B. abortus* bv. 4	NP
15	*Brucella* spp.	*B. abortus* bv. 1	Field strain
16	*Brucella* spp.	*B. abortus* bv. 1	Field strain
17	*Brucella* spp.	*B. abortus* bv. 4	NP

NP: Not performed (mAMOS-PCR not applied to *B. abortus* biovar 4 isolates, as vaccine strains belong exclusively to biovar 1).

**Table 6:** Distribution of results from serological tests (RB and SAT-EDTA) in pigs, depending on the sampling location (slaughterhouse) and origin of sampled animals.

Province Location Slaughterhouse	Slaughterhouse	Province of Animal Origin	Sex of Animals Sampled	Number of Samples	Serological Test Results				Microbiological Isolates
					RB	SAT-EDTA	RB and SAT-EDTA	Positives to Serology	
Carchi				566					
	San Gabriel			160					
		Carchi		160					
			Female	76	0	0	0	0	0
			Male	84	1	0	0	1	0
	Tulcán			406					
		Carchi		406					
			Female	203	0	0	0	0	0
			Male	203	0	1	0	1	0
Imbabura				365					
	Atuntaqui			114					
		Imbabura		114					0
			Female	33	1	1	0	2	0
			Male	81	0	2	0	2	0
	Ibarra			251					0
		Imbabura		251					0
			Female	173	0	0	0	0	0
			Male	78	0	0	0	0	0
Pichincha				119					
	Cayambe			119					
		Imbabura		7					
			Female	5	0	0	1	1	0
			Male	2	0	0	0	0	0
		Pichincha		112					
			Female	69	0	4	0	4	0
			Male	43	0	0	0	0	0
Total				1050	2	8	1	11	0

## Data Availability

The original contributions presented in this study are included in the article. Further inquiries can be directed to the corresponding author(s).
